# Intraoperative near-infrared fluorescent imaging during robotic operations

**DOI:** 10.1590/S1679-45082016MD3658

**Published:** 2016

**Authors:** Antonio Luiz de Vasconcellos Macedo, Vladimir Schraibman

**Affiliations:** 1 Hospital Israelita Albert Einstein, São Paulo, SP, Brazil.

**Keywords:** Cholecystectomy, Colectomy, Fluorescence, Robotics, Intraoperative period, Indocyanine green

## Abstract

The intraoperative identification of certain anatomical structures because they are small or visually occult may be challenging. The development of minimally invasive surgery brought additional difficulties to identify these structures due to the lack of complete tactile sensitivity. A number of different forms of intraoperative mapping have been tried. Recently, the near-infrared fluorescence imaging technology with indocyanine green has been added to robotic platforms. In addition, this technology has been tested in several types of operations, and has advantages such as safety, low cost and good results. Disadvantages are linked to contrast distribution in certain clinical scenarios. The intraoperative near-infrared fluorescent imaging is new and promising addition to robotic surgery. Several reports show the utility of this technology in several different procedures. The ideal dose, time and site for dye injection are not well defined. No high quality evidence-based comparative studies and long-term follow-up outcomes have been published so far. Initial results, however, are good and safe.

## RESUMO

A identificação intraoperatória de certas estruturas anatômicas, por seu tamanho ou por elas serem ocultas à visão, pode ser desafiadora. O desenvolvimento da cirurgia minimamente invasiva trouxe dificuldades adicionais, pela falta da sensibilidade tátil completa. Diversas formas de detecção intraoperatória destas estruturas têm sido tentadas. Recentemente, a tecnologia de fluorescência infravermelha com verde de indocianina foi associada às plataformas robóticas. Além disso, essa tecnologia tem sido testada em uma variedade de cirurgias, e suas vantagens parecem estar ligadas a baixo custo, segurança e bons resultados. As desvantagens estão associadas à má distribuição do contraste em determinados cenários. A imagem intraoperatória por fluorescência infravermelha é uma nova e promissora adição à cirurgia robótica. Diversas séries mostram a utilidade da tecnologia em diferentes procedimentos. Dose ideal, local e tempo da injeção do corante ainda não estão bem estabelecidos. Estudos comparativos de alta qualidade epidemiológica baseados em evidência ainda não estão disponíveis. No entanto, os resultados iniciais são bons e seguros.

## INTRODUCTION

Intraoperative identification of certain anatomical structures may be challenging. Small parts, such as lymph nodes, relevant in an oncologic operation, or structures visually occult to the eye may be left behind due to its intraparenchymal situation or low contrast with surrounding environment that may be damaged or require a great deal of dissection to be located.^(1)^ The development of minimally invasive surgery brought countless advantages over conventional surgery. However, disadvantages are also associated to these procedures and the lack of complete tactile sensitivity brings additional difficulties to the identification of the aforementioned structures.

Many different forms of intraoperative mapping have been tried. Colorization by the injection of vital dyes (India ink, methylene blue, patent blue, indigo carmine etc.),^(2)^ radioisotope tracing,^(3)^ intraoperative imaging (ultrasound, tomography etc.),^(4)^ fluorescence^(1)^ or combination of pre-acquired radiologic and intraoperative images (hybrid imaging)^(5)^ are some of the available methods.

Recently, near-infrared fluorescence imaging technology with indocyanine green has been added to robotic platforms and become commercially available since 2011. In 2014, it was cleared by the Food and Drug Administration (FDA) and Brazilian regulatory agency (ANVISA - *Agência Nacional de Vigilância Sanitária*). The technique consists of bolus injection of the drug in peripheral veins (for hepatobiliary imaging due to the biliary excretion of the substance), regional vessels (for vascular anatomy identification), or submucosal or subserosal (for lymph nodes identification or tumoral tattooing).^(6)^ The safe dose recommended for a standard diagnostic procedure is 0.1 to 0.5mg/kg, which is stimulated with infrared laser light responding with a highly intense fluorescent signal that is captured by special cameras in combination or not with visible spectrum images.^(7)^

**Figure 1:**
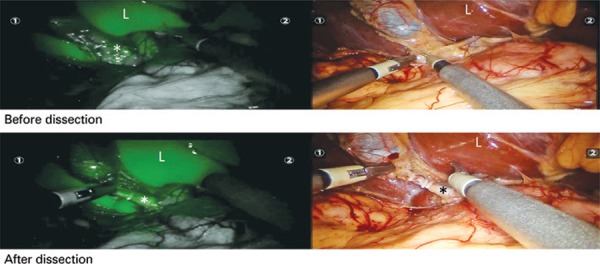
Biliary anatomy revealed by intraoperative near-infrared fluorescent imaging during a single-port robotic cholecystectomy in a 42-year-old female patient. Indocyanine green dye was injected intravenously 30 minutes before the image was taken. Biliary tree is identified in green even before dissection because of fluorescence (top, left). Biliary anatomy is still occult at the corresponding image in visible light spectrum (top, right). The liver parenchymal contrast can be seen as well. After dissection of the cystic duct (bottom), biliary anatomy is even clearer

**Figure 2:**
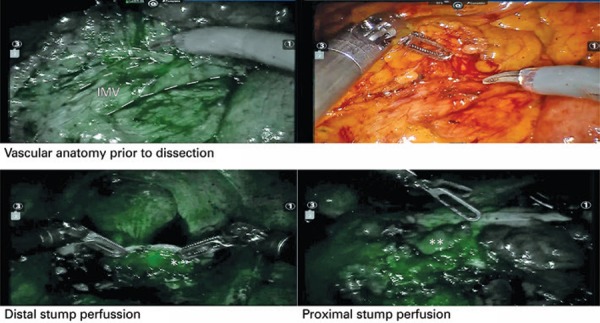
Vascular anatomy revealed by intraoperative near-infrared fluorescent imaging during a robotic sigmoidectomy. Inferior mesenteric vessels and branches (dotted line) were ready identified prior to dissection with indocyanine green dye (top). After dissection, an adequate perfusion of the distal stump (bottom, left) was confirmed and a highly vascularized portion of the colon was selected for the anastomosis based on the microvascularization seen after fluorescence with indocyanine green dye injected (bottom, right)

The technology has been tested in several types of surgical procedures from the identification of structures for retrieval, such as lymph node harvesting in oncologic surgeries or endometriosis foci,^(8,9)^ to prevent organ injuries, such as ureter or biliary ducts. This prevention is made by contrasting these structures^(6,10)^ to blood vessels anatomy and their identification, allowing partial resection of solid organs or anastomosis in better vascularized spots,^(6,7)^ however, the knowledge of this system is still incipient. Most studies published so far are small series or case reports. Our personal experience is limited to an elective cholecystectomy (Figure 1) and a colectomy (Figure 2), both performed at the *Hospital Israelita Albert Einstein*.

*Cystic duct; L: liver.

*Distal stumps (rectum). ** Well perfused proximal level of resection.

IMV: inferior mesenteric vessels.

Advantages of the method are an apparent decrease in intraoperative time, an increase in the number of lymph nodes harvested, and correct identification of biliary and vascular anatomy.^(6,7)^ Costs are low, the equipment does not have a prohibitively cost and the drug is inexpensive.^(7)^ The dye (indocyanine) has few side effects, low toxicity and allergic reactions.^(6)^ The drug may be used to up to 6 hours after reconstitution.^(7)^

Disadvantages are linked to the time for the dye to be distributed in emergency situations, decrease accuracy in obese individuals,^(8)^ after radiotherapy,^(7)^ in inflamed tissues^(7)^ and with preoperative injection of the dye.^(7)^ Probably, the future knowledge of ideal dose, time and site for dye injection may improve these scenarios.

The intraoperative near-infrared fluorescent imaging is a new and promising addition to robotic surgery. Several reports show the useful of this technology in several different procedures. The ideal dose, time and site for dye injection are not well defined. No high quality evidence-based comparative studies and long-term follow-up have been published so far. The initial results, however, are good and safe.
